# Synthesis, characterization, antitumor potential, and investigation of mechanism of action of copper(ii) complexes with acylpyruvates as ligands: interactions with biomolecules and kinetic study†

**DOI:** 10.1039/d2ra05797b

**Published:** 2022-10-26

**Authors:** Nenad Joksimović, Jelena Petronijević, Snežana Radisavljević, Biljana Petrović, Kristina Mihajlović, Nenad Janković, Emilija Milović, Dušan Milivojević, Bojana Ilić, Ana Djurić

**Affiliations:** University of Kragujevac, Faculty of Science, Department of Chemistry Radoja Domanovića 12 34000 Kragujevac Serbia nenad.joksimovic@pmf.kg.ac.rs; University of Kragujevac, Institute for Information Technologies Kragujevac, Department of Sciences Jovana Cvijića bb 34000 Kragujevac Serbia; Vinča Institute of Nuclear Science University of Belgrade P.O. Box 522 11001 Belgrade Serbia; Clinic for Endocrinology, Diabetes and Metabolic Diseases, University Clinical Centre of Serbia Belgrade Serbia; Institute of Oncology and Radiology of Serbia Pasterova 14 11000 Belgrade Serbia

## Abstract

Considering the urgency of finding a cure for vicious diseases such as tumors, we have synthesized and characterized a small series of new copper(ii) complexes with biologically important ligands such as acylpyruvate. In addition to this, we used another four copper(ii) complexes, with ligands of the same type to examine the antitumor potential. The antitumor potential of the copper(ii) complexes was examined on three tumor cell lines and one normal human cell line using the MTT assay. All seven tested complexes showed very good cytotoxic effects. Two copper complexes that showed the best antitumor potential were selected for further testing that showed the best potential for potential application in the future. The mechanism of activity of these complexes was examined in detail using tests such as cell cycle, ROS level, oxidative DNA damage, and proteins related to hypoxia analysis. In addition, we examined the binding abilities of these complexes with biomolecules (Guo, Ino, 5′-GMP, BSA, and DNA). The results showed that the tested compounds bind strongly to DNA molecules through intercalation. Also, it has been shown that the tested compounds adequately bind to the BSA molecule, which indicates an even greater potential for some future application of these compounds in clinical practice.

## Abstract 2

Considering the urgency of finding a cure for vicious diseases such as tumors, we have synthesized and characterized a small series of new copper(ii) complexes with biologically important ligands such as acylpyruvate.

## Graphical abstract

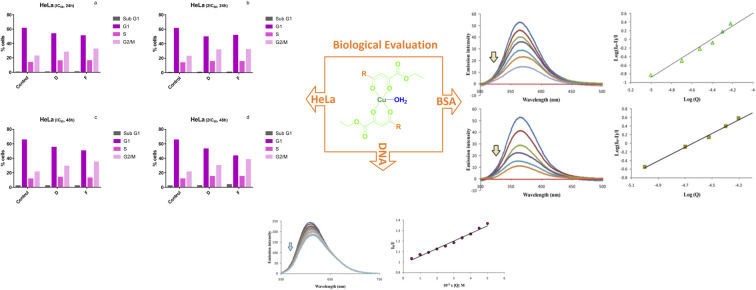

## Introduction

**Fig. 1:**
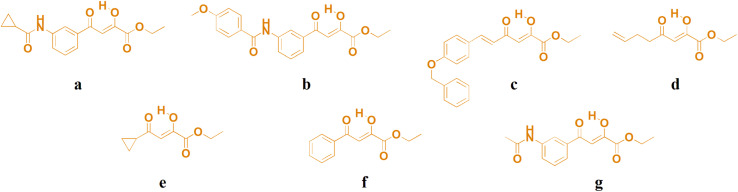
Structures of ligands a–g.

People have been struggling with many vicious and deadly diseases from ancient times for which there was no adequate cure. With the development of medical chemistry and science in general, we have discovered a large number of drugs for the treatment of these vicious diseases. However, even in the modern world, there are some difficult-to-treat and widespread diseases for which there is no adequate drug therapy. It can be freely said that cancer is one of those diseases and is one of the most common causes of death today. Therefore, research in this area is of great importance, and finding a cure for this vicious disease is vital to humanity.^1–5^ With the discovery of cisplatin, significant progress has been made in the struggle against various types of cancer.^6^ However, cisplatin is not an ideal drug due to several side effects such as nephrotoxicity, neurotoxicity, ototoxicity, and resistance to certain types of tumors.^7–10^ All this has forced the scientific community around the world to discover a new drug that would have a similar effect as cleavage but with fewer side effects. Numerous studies have been conducted with different strategies.^11–15^ One of the most important is the replacement of platinum metal ions with some other metal that shows less toxicity or, in an even better case, with some essential metal.^16–18^ Keeping in mind the previous and knowing that copper is an essential element with numerous biological functions in the human organism, as well as our previous experiences with copper compounds, we came up with the idea to examine the antitumor potential of a series of copper(ii) complexes. A small series of new copper(ii) complexes with biologically significant ligands such as acylpyruvate (Fig. 1.) were synthesized for these studies. In addition to this, the same investigations were also performed on four copper(ii) complexes, which we previously synthesized with ligands of the same type.^19^ All complexes were tested on four cell lines. A mechanism of antitumor activity was performed and interactions with biomacromolecules such as deoxyribonucleic acid (DNA) and bovine serum albumin (BSA). Studies of the interaction of a molecule and DNA are crucial for its potential antitumor activity.^20,21^ In addition, although a potential drug exhibits exceptional biological potential, it is important to examine its binding capacity for transport proteins such as serum albumins.^22,23^ One of the prominent roles of bovine serum albumin is the transport of endogenous and exogenous ligands and drugs in the bloodstream. Investigations of the binding capacity of a potential drug to BSA are very important because of the similar structure of this molecule to human serum albumin.

## Results and discussion

### Chemistry

**Scheme 1:**
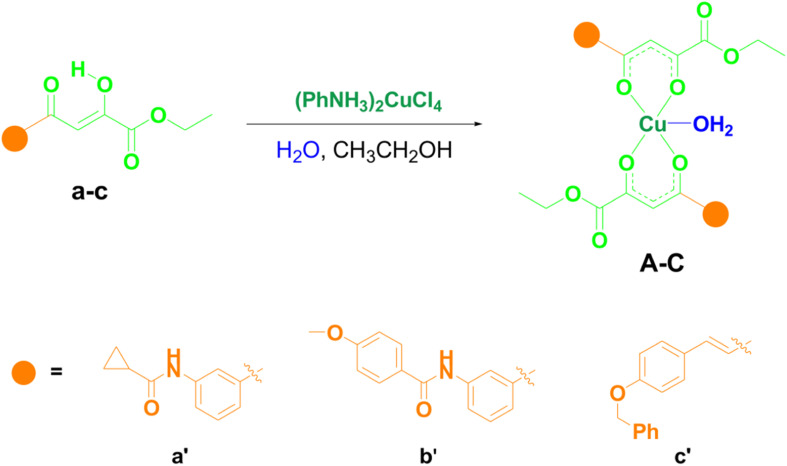
Synthetic procedures for the synthesis of novel copper(ii) complexes A–C.

Three new copper(ii) complexes were synthesized in a simple reaction between different substituted *O*,*O*-bidentate ligands acylpyruvates and (PhNH_3_)_2_CuCl_4_ in ethanol at room temperature by the procedure we described earlier.^24^ In terms of the functional groups, these specific structures of ligands were chosen for the investigations since some of its derivatives have shown good biological potential in some of our previous studies.^19,24^ The synthesis of novel copper(ii) complexes A–C with acylpyruvates a–c is shown in Scheme 1. All ligands were previously synthesized.^25^ As can be seen, in the reaction between acylpyruvate a–c and (PhNH_3_)_2_CuCl_4_ in a ratio of 2 : 1 we obtained three novel copper(ii) complexes with square-pyramidal geometry, where the base of the pyramid is engaged by four oxygen from two acylpyruvate ligands. At the same time, the fifth coordination place is occupied by a molecule of water, the molecular geometry confirmed using X-ray diffraction and electron paramagnetic resonance analysis in our previous study.^24^ All the complexes were obtained in high yields.

**Fig. 2:**
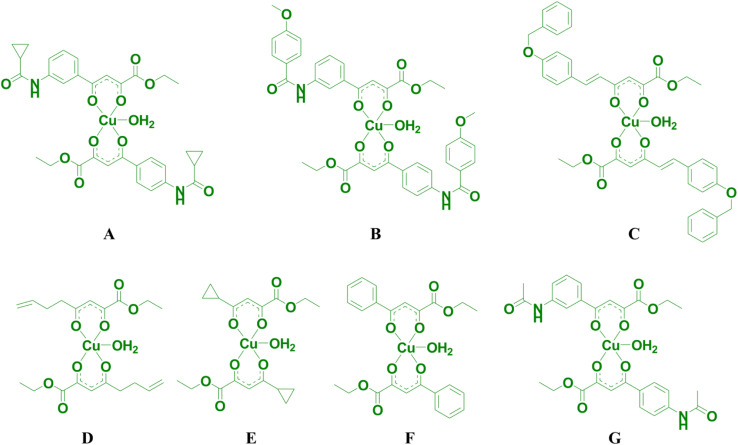
Structures of the tested copper(ii) complexes A–G.

The novel copper complexes were characterized using UV-vis, IR, EPR, and MS spectroscopy, and elemental analysis. The IR spectra of complexes A–C displayed bands near 1570 and 1510 cm^−1^ that correspond to *v*(C=C) (coupled with *ν*(C=O)) and *ν*(C=O) (coupled with *ν*(C=C)), respectively. Therefore, in IR spectra of complexes, the *v*(C=O) band is negatively shifted by approximately 15 cm^−1^, compared to the corresponding spectra of ligand. The presence of the bands near 1270 cm^−1^, which is intense, in the spectra of ligands a–c occurs due to bending O–H vibrations in the plane.^26,27^ The nonexistence of these bands in the spectra of complexes A–C is the consequence of the coordination of the ligands to copper(ii) ions. Lastly, three novel A–C and four previously obtained copper(ii) complexes D–G (ref. 19) (Fig. 2) were used to study their antitumor potential.

### EPR study

Magnetic resonance techniques are very sensitive inner probes into the structure and properties of materials. g-Factor and the hyperfine interaction are very sensitive to the environment, coordination, and symmetry around Cu^2+^. Orbital (2L + 1) degeneracy is removed according to the symmetry formed by surrounding ligands. The spin–orbit interaction between the spin only orbital quenched ground state (*E*_0_) and the excited states (*E*_n_) reintroduces a little orbital contribution and the *g* is anisotropic with values that deviate from 2.0023.^19,28^
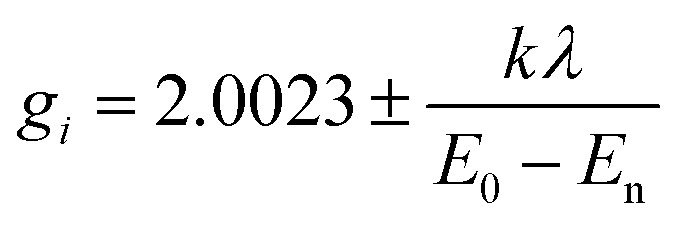
*i* = *x*, *y*, *z*, *λ* is the spin–orbit coupling constant, while *k* defines the degree of orbital mixing.

**Fig. 3:**
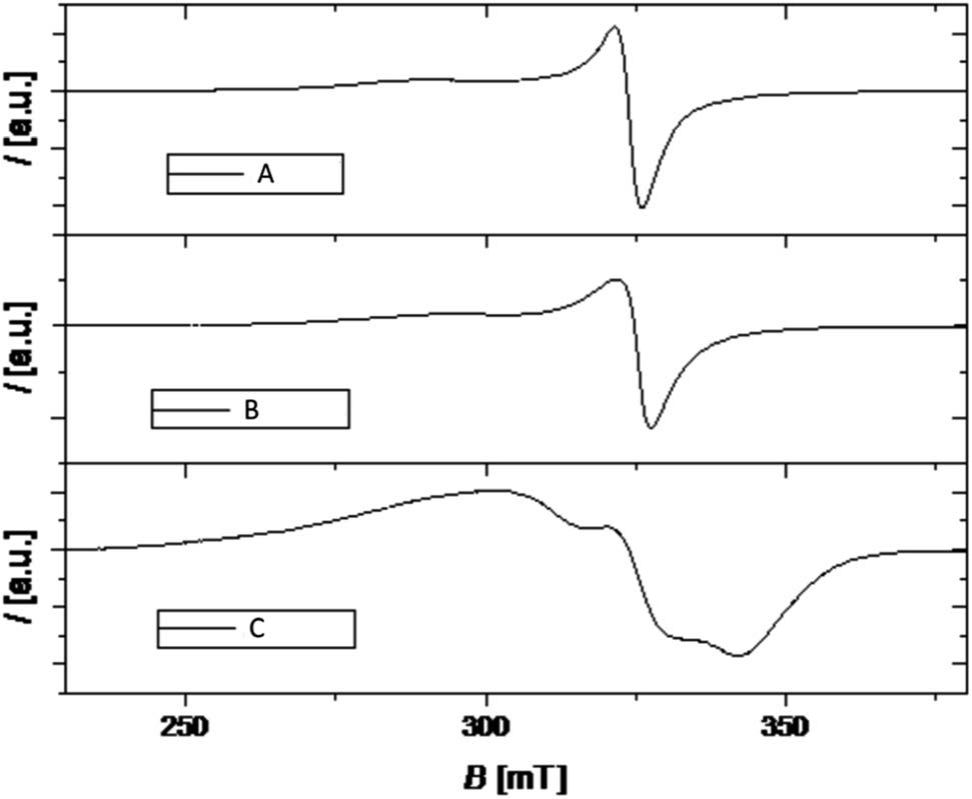
EPR spectra of copper(ii) complexes A–C (black lines).

The *g*-factor reflects the nature of the ground state. For elongated octahedral, square pyramidal, or square planar the ground state is d_*x*^2^−*y*^2^_ orbital the EPR spectrum is axial and *g*_‖_ > *g*_⊥_ > *g*_e_ is expected (*g*_‖_ = *g*_*z*_, *g*_⊥_ = *g*_*x*_ = *g*). When the geometry is compressed octahedral or trigonal bipyramidal the ground state is d_*z*^2^_ and *g*_⊥_ > *g*_‖_ = *g*_e_ is expected. In intermediate situations, a rhombic spectrum, *g*_*x*_ > *g*_*y*_ > *g*_*z*_, is expected. The EPR spectra of compounds A–C are presented in Fig. 3. For such complexes, a parameter *R* can indicate the predominance of the d_*z*^2^_ or d_*x*^2^−*y*^2^_ orbital. *R* = (*g*_*y*_ − *g*_*z*_)/(*g*_*x*_ − *g*_*y*_). If *R* > 1 (*R* < 1) the greater contribution to the ground state arises from the d_*z*^2^_ (d_*x*^2^−*y*^2^_).^19,28^

The values of *g*-tensor are presented in Table 1. The *g* values for samples A and B resemble relation *g*_‖_ > *g*_⊥_ > *g*_e_ and since the coordination number of investigated complexes around Cu ion is five, we conclude that the geometry around Cu is square pyramidal.

**Table 1:** EPR spectra *g* values

Sample	*g*_1_	*g*_2_	*g*_3_
A	2.076	2.079	2.36
B	2.068	2.069	2.34
C	1.97	2.08	2.33

Sample C shows noticeable rhombic symmetry but calculated *R* = 0.44 (<1) is indicative of d_*x*^2^−*y*^2^_ ground state and square pyramidal geometry.

### Anticancer evaluation

The molecular structures of the copper(ii) complexes (A–G) that are used for testing the anticancer potential are shown in Fig. 2. All the complexes are tested on one normal (human embryonic lung fibroblast (MRC-5)) and three cancer (human cervical adenocarcinoma (HeLa), human colon adenocarcinoma (LS174T), and human lung adenocarcinoma (A549)) cell lines.

### Cytotoxic activity

The seven copper complexes were assessed for cytotoxic activity (72 h of incubation), against one normal cell line (MRC-5) and three cancer cell lines (HeLa, LS174T, and A549) (Table 2). According to the obtained results compounds B, D, and F showed the most potent cytotoxic activity against all tested malignant cell lines. While compounds E and G showed significant reductive activities against A549 and MRC5 cell lines. For further biological assessment, compounds D and F were selected. We also examined the cytotoxic activity of chosen compounds, D and F, at 24 h of treatment, against HeLa cells. Our results have shown that the IC_50_ value for substance F decreases with the time of exposure, while IC_50_ values for substance D are similar at 24 h and 72 h treatment (Table 3).

**Table 2:** Cytotoxic activity (IC_50_ values) of copper(ii) complexes A–G against MRC5, LS174T, A549, and HeLa cells at 72 h of treatment^a^

Compounds	MRC5 IC_50_ (μM)	LS174T IC_50_ (μM)	A549 IC_50_ (μM)	HeLa IC_50_ (μM)
A	86.65 ± 1.40	>200	74.65 ± 8.84	182.17 ± 4.95
B	11.44 ± 1.8	19.61 ± 2.73	12.90 ± 1.24	15.24 ± 2.33
C	45.07 ± 1.79	47.00 ± 0.63	48.55 ± 2.65	46.37 ± 4.5
D	15.45 ± 2.70	35.55 ± 2.89	14.94 ± 1.96	17.86 ± 0.15
E	18.85 ± 1.11	39.95 ± 1.31	13.98 ± 3.17	26.79 ± 1.5
F	13.12 ± 2.64	22.93 ± 2.33	12.74 ± 1.82	15.41 ± 1.23
G	20.77 ± 3.5	24.98 ± 2.69	15.67 ± 0.23	36.12 ± 0.73
cisPt	9.35 ± 1.29	5.54 ± 1.03	13.21 ± 0.89	4.91 ± 0.74

a IC_50_ values (μM) were expressed as the mean ± SD determined from the results of the MTT assay in three independent experiments.

**Table 3:** Cytotoxic activity (IC_50_ values) of copper compounds against HeLa cells at 24 h of treatment^a^

Compounds	HeLa IC_50_ (μM)
D	15.41 ± 1.23
F	17.86 ± 0.15

a IC_50_ values (μM) were expressed as the mean ± SD determined from the results of the MTT assay in three independent experiments.

By examining the dependence of the activity on the structure of the tested compounds, we came to the conclusions that the copper(ii) complexes showed the best activity when the allyl or phenyl group was located on the acylpyruvate residue. Complexes with ligands that contained cyclopropyl, 3-acetamidophenyl or 3-(4-methoxybenzamido)phenyl groups in a row also showed good activity. The weakest activity was shown by complexes with ligands whose residues were 3-(cyclopropanecarboxamido)phenyl or 4-benzyloxystyryl groups.

### Cell cycle analysis

**Fig. 4:**
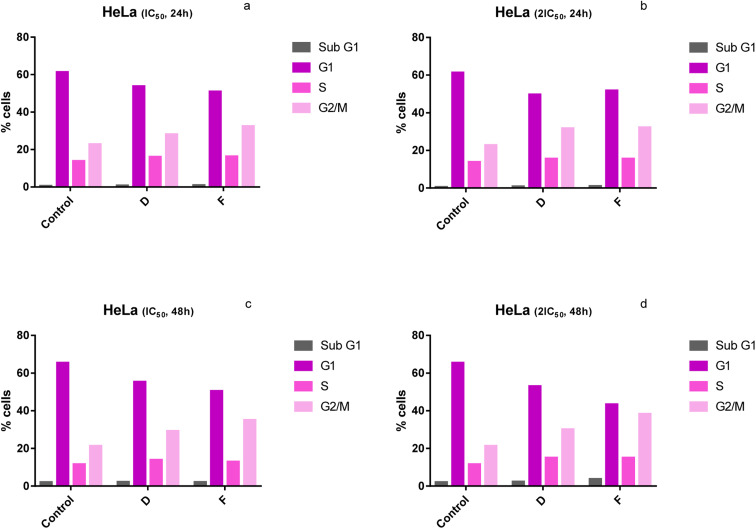
(a–d) Changes in the cell cycle of HeLa cells treated with tested compounds (concentrations correspond to IC_50_ and 2IC_50_) at 24 h and 48 h of treatment.

The effect of selected newly synthesized substances on the cell cycle distribution was evaluated to better understand the mechanism of their cytotoxicity against HeLa cells. The distribution of HeLa cells (%) in different phases of the cell cycle treated with tested compounds is presented in Table 4. The results presented in Fig. 4 correspond to the distribution of the cell cycle of HeLa cells treated with compounds D and F (IC_50_ and 2IC_50_) for 24 h and 48 h. Assessment of the changes in the cell cycle of HeLa cells upon incubation with two doses, IC_50_ and 2IC_50_, of tested compounds (D and F) for 24 h and 48 h revealed that tested substances cause the increase in the percentage of HeLa cells in G2/M phase and the decrease in G1 phase, compared to control (untreated cells) (Fig. 4).

**Table 4:** Distribution of HeLa cells (%) in different phases of the cell cycle treated with tested compounds (concentrations corresponding to IC_50_ and 2IC_50_) at 24 h and 48 h of treatment

Cell cycle phase	Sub-G1	G1	*S*	G2/M
**Treatment for 24 h**				
CONTROL	0.48	61.29	13.85	22.77
**D** (IC_50_)	0.72	53.69	16.01	28.11
**D** (2IC_50_)	0.86	49.63	15.58	31.73
**F** (IC_50_)	0.87	50.91	16.24	32.34
**F** (2IC_50_)	0.96	51.77	15.58	32.16

**Treatment for 48 h**				
CONTROL	2.05	65.40	11.58	21.31
**D** (IC_50_)	2.17	55.28	13.90	29.22
**D** (2IC_50_)	2.31	53.03	15.03	30.11
**F** (IC_50_)	2.08	50.44	12.93	35.03
**F** (2IC_50_)	3.70	43.34	15.06	38.32

Our results have demonstrated that after the 24 h-exposure to compounds D and F, the percentage of HeLa cells in the G1 phase was 50.4% and 55.3%, respectively, for IC_50_ 51.8% and 53%, respectively, for 2IC_50_, compared to 61.3% (control) (Fig. 4a and b). While, for 48 h-exposure to compounds D and F the observed proportions of cells in the G1 phase were 50.4% and 55.3%, respectively, for IC_50_ and 43.3% and 53%, respectively, for 2IC_50_, compared to 65.4% (control) (Fig. 3c and d). We have shown that treatment with substances D and F caused a significant increase in the percentage of HeLa cells in the G2/M phase of the cell cycle (Fig. 4). After the treatment of 24 h to compounds D and F percentage of HeLa cells in the G2/M phase, phases were 32.4% and 28.1%, respectively, for IC_50_ and 32.2% and 31.7%, respectively, for 2IC_50_, compared to 22.8% (control) (Fig. 4a and b). While, after the 48 h-treatment of compounds D and F the observed concentrations of cells in the G2/M phase were 35% and 29.2%, respectively, for IC_50_ and 38.3% and 30.1%, respectively, for 2IC_50_, compared to 21.3% (control) (Fig. 4c and d).

Cell growth and division are precisely regulated by complex signaling pathways that affect the cell cycle to determine the cell's fate. Many different mechanisms influence the normal cell cycle. Cancer cells divide constantly and excessively thanks to cancer-related mutations that influence cell cycle control mainly by compromising the ability of cells to exit the cell cycle.^29,30^ Previous studies have revealed that many anti-cancer drugs have caused the arrest of the cell cycle at a certain phase.^31^ In the present study, the results indicate that tested compounds caused cell cycle arrest of HeLa cells at the G2/M phase. Therefore, tested newly synthesized copper compounds have the potential to achieve an anticancer effect. The cell cycle arrest at the G2/M phase indicates that DNA damage cannot be repaired and the cell undergoes apoptosis.^32^

### Analyzes of ROS level

**Fig. 5:**
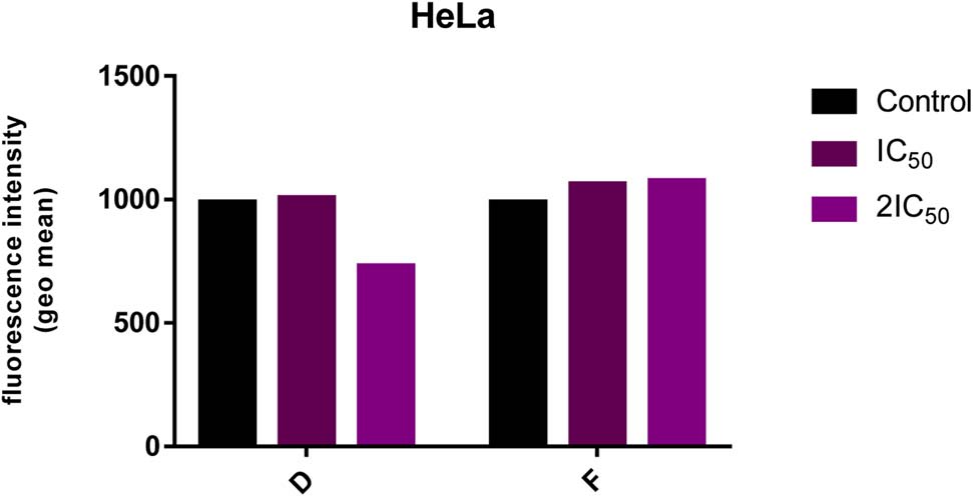
Level of endogenous ROS in the HeLa cells following the 24 h-exposure to tested compounds (IC_50_ and 2IC_50_). The intensity of fluorescence corresponds to the level of ROS.

The endogenous ROS level in HeLa cells after 24 h-treatment with 2IC_50_ of compound D was reduced compared to ROS levels in the control group, while treatment with compound F did not change the ROS level in treated HeLa cells (Fig. 5).

Elevated ROS levels are connected to several pathologic conditions, including cancer, and they are involved in different signaling pathways and the triggering of DNA mutations. One of the approaches in cancer treatment is based on lowering ROS levels in cancer cells. According to this therapeutic approach,^33^ compound D could achieve a potential anticancer effect by reducing the level of ROS in the treated malignant cells. This possibility should be further investigated.

For normal cell survival, maintaining the physiological concentration of ROS is necessary. Overproduction of ROS promotes cell proliferation and induces the malignant transformation of normal cells through different signaling pathways.^33^ Therefore, scavenging elevated ROS levels prevent the formation of early neoplasia.^34^ This is in line with the recent studies that have shown an increase in tumor development and metastasis in mouse models treated with vitamin E.^35^ On the other hand, abnormally ROS production specifically kills cancer cells, which also can be used as a promising anticancer therapeutic strategy.^33^

### Analyzes of DNA damage

**Fig. 6:**
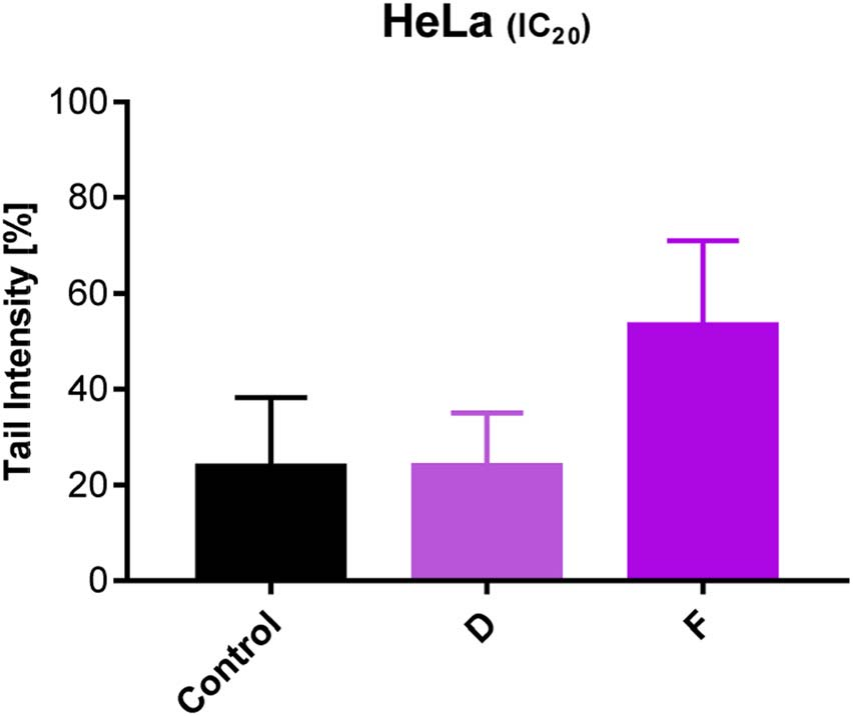
Effect of 24 h-treatment with tested compounds on DNA damage in HeLa cells using the Comet assay.

After 24 h treatment with subtoxic IC_20_ concentrations of selected compounds (D and F), the degree of DNA damage in HeLa cells was evaluated. Our results point out that exposure to compound F caused DNA damage, which is significantly higher compared to the values in the control group (untreated cells) (*p* < 0.001) (Fig. 6).

### Analyzes of western blot

**Fig. 7:**
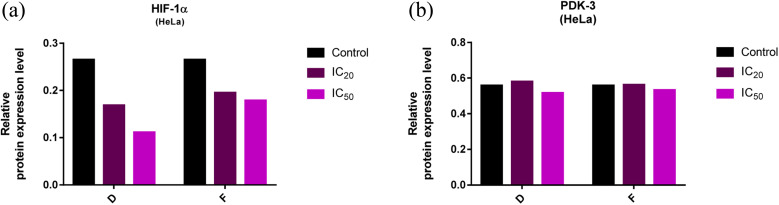
(a and b). Expression of HIF-1α and PDK-3 in HeLa cells after 24 h treatment with tested compounds (concentrations correspond to IC_20_ and IC_50_).

**Fig. 8:**
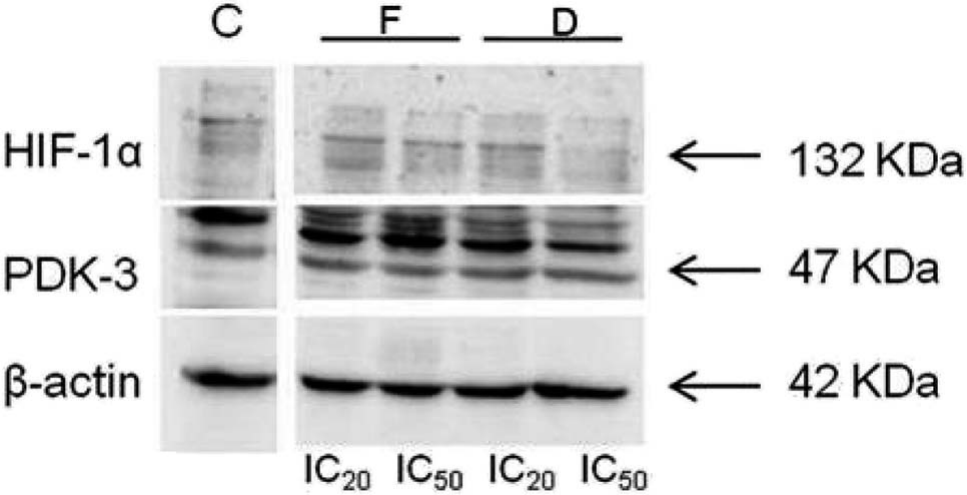
Immunoblots for HIF-1α, PDK-3, and β-actin of HeLa whole-cell lysates after 24 h treatment with tested compounds (concentrations correspond to IC_20_ and IC_50_).

The expression levels of PDK-3 and HIF-1α proteins (normalized to β-actin) in HeLa cells treated with compounds D and F, and untreated (control) HeLa cells are presented in Fig. 7 and 8. HeLa cells treated with compounds D and F at concentration IC_50_ demonstrated lower expression level of HIF-1α protein in comparison to HeLa cells treated with concentration IC_20_ and to control HeLa cells. Irrespective of treatment, HeLa cells similarly expressed PDK-3 protein.

### Binding properties of compounds D and F with bovine serum albumin

**Fig. 9:**
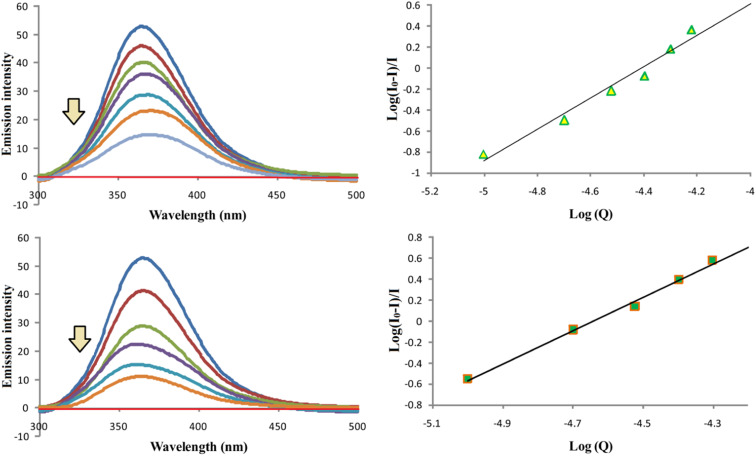
Binding mode of copper complexes D and F with BSA. Emission spectra of BSA in the absence (the most intense blue lines) and in the existence of copper complexes D and F. Red lines match up to the solution (buffer + compound). [BSA] = 10.0 μM; [D] = 0.0–30.0 μM, [F] = 0.0–25.0 μM; pH = 7.4; *λ*_ex_ = 280 nm.

Compounds D and F were used to investigate the binding properties of these compounds to BSA. The fluorescent titration method was used for these investigations. All the measurements were done according to the previous procedure we used.^36^ We measured the fluorescence intensity of BSA in the absence (most intense blue line) and in the presence of compounds D and F (Fig. 9). The emission spectra are recorded in the wavelength range from 300 to 500 nm, while *λ*_ex_ was set at 280 nm. The molar ratios of BSA with compound D followed orders: 1 : 0 (control), 1 : 0.5, 1 : 1, 1 : 1.5, 1 : 2, 1 : 2.5, and 1 : 3, while for F followed orders: 1 : 0 (control), 1 : 0.5, 1 : 1, 1 : 1.5, 1 : 2, and 1 : 2.5. Based on the obtained results, it can be seen that fluorescence intensity of BSA decreases with increasing concentration of the compounds D and F, which occurs as a result of the formation of the BSA-D or F complex. Based on the obtained results, by observing the dependence of log(*I*_0_ − *I*)/*I* on log(*Q*) (Fig. 9), binding parameters were calculated. Where *I*_0_ is the fluorescence intensity of BSA in the absence of compounds D and F, *I* is the fluorescence intensity of BSA in the presence of compounds D and F, and *Q* is the concentration of compounds D and F. Based on the obtained linear dependence and the equation log(*I*_0_ − *I*/*I*) = log *K*_a_ + *n* log[*Q*], we calculated the *n* and *K*_a_ binding parameters of compounds D and F to BSA, where *n* is the number of binding spots per BSA molecule, and *K*_a_ is the binding constant of the tested compounds to BSA. The obtained *K*_a_ and *n* values are shown in Table 5. Obtained *K*_a_ values are in line with the fact that our compounds have the appropriate capabilities for future use in clinical practice because they appropriately bind to BSA.^37^ On the other hand, the number *n* revealed that compounds D and F bind to BSA molecules in a 1.5 : 1 molar ratio.

**Table 5:** Binding parameters *K*_a_ and *n* and the correlation coefficient (*R*) for binding mode of D and F with BSA

Compound	*K*_a_ [M^−1^]	*n*	*R*
D	(3.8 ± 0.2) × 10^6^	1.5	0.986
F	(5.2 ± 0.2) × 10^6^	1.5	0.997

### Investigations of binding properties of compound F with DNA

**Fig. 10:**
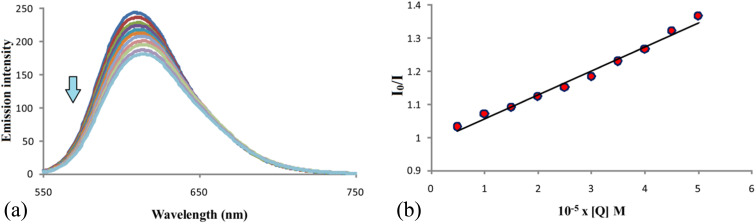
(a) Emission spectra of EB–DNA complex in the absence of F (the most intense blue line), and in the presence of F. [EB] = 50 μM, [DNA] = 50 μM; [F] = 0-50 μM; pH = 7.4; *λ*_ex_ = 500 nm. (b) Plots of *I*_0_/*I versus* concentration of F ([*Q*]).

The fluorescent titration method, a very accurate and reliable method, was used to investigate the interactions of compound F that showed the best antitumor activity with DNA. For these studies, we used ethidium bromide (EB), one of the most commonly used intercalators for interactions of this type.^38^ The fluorescence intensities of the EB–DNA complex in were measured in the absence of compound F (the most intense blue line) and in the presence of the tested compound (Fig. 10). Fluorescence intensities were observed at wavelengths from 550 to 750 nm, and *λ*_ex_ was set at 500 nm. Investigations have shown that with increasing concentration of compound F, the fluorescence intensity of the EB–DNA complex decreases. In addition, the maximum wavelength of the EB–DNA at 609 nm was red-shifted. Based on the above, it can be concluded that compound F partially substitutes EB from the EB–DNA complex and interacts with DNA *via* intercalation. Further, by examining the dependence of *I*_0_/*I* on the concentration *Q* of the compound and using the Stern–Volmer equation,^38^ some binding parameters of compound F for DNA were calculated, such as *K*_sv_ and *k*_q_ (Table 6). *I*_0_ is the fluorescence intensity of EB–DNA in the absence of compound F, *I* is the fluorescence intensity of EB–DNA in the presence of compound F, *Q* is the concentration of compound F, *K*_sv_ is the Stern–Volmer constant and *k*_q_ is the bimolecular quenching rate constant. The obtained *K*_sv_ constant of 7.3 × 10^3^ shows that the compound binds strongly to DNA molecules through intercalation.^39^

**Table 6:** Binding parameters (*k*_q_, *K*_sv_, and correlation coefficient (*R*)) of F with DNA

Compound	*k*_q_ [M^−1^ s^−1^]	*K*_sv_ [M^−1^]	*R*
F	(7.3 ± 0.1) × 10^11^	(7.3 ± 0.1) × 10^3^	0.993

### Kinetic measurements for compound F

It is already known that the copper(ii) ion belongs to the group of “borderline” Lewis acid, so it has a high affinity to bind with nitrogen-, oxygen- and sulfur donors, depending on the coordination number. The presence of different mono-, bi-, tri-, or tetradentate inert ligands in the structure of copper(ii) complexes has a huge effect on their substitution reactions. Also, the nature of the leaving group is strongly connected with the substitution rate.^40,41^ This work is focused on the following substitution reactions between copper(ii) complex and biomolecules such as Guo, Ino, or 5′-GMP. The aim is to reveal the complex's capability to react with nucleophiles, especially with 5′-GMP, which is the constituent of DNA (due to the fact that this complex can bind to DNA with DNA-binding constant *K*_b_ = 7.3 × 10^3^).

**Fig. 11:**
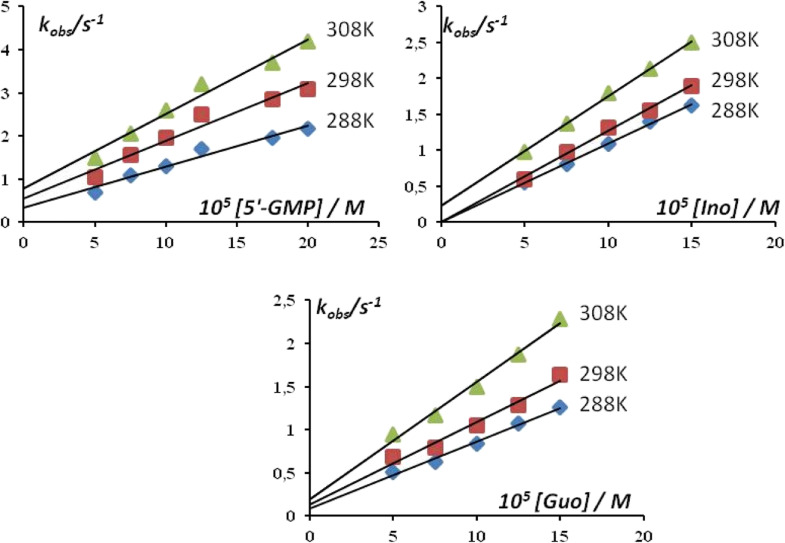
Pseudo-first-order rate constant *k*_obs_ as a function of nucleophile concentration and temperature for the substitution reactions in Hepes buffer (pH = 7.2, 25 mM Hepes).

**Scheme 2:**
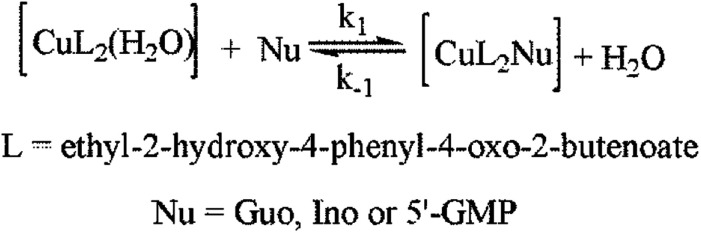
Substitution reaction of studied Cu(ii) complex F.

Substitution reactions of five-coordinate copper(ii) complex with nucleophiles (Guo, Ino, or 5′-GMP) were very fast to be followed by conventional spectrophotometry (Fig. 11). So, these reactions were studied by the “stopped-flow” technique, mixing the same volumes of complex and nucleophile solutions directly in the instrument. To achieve the pseudo-first-order conditions for all experiments, the concentration of the nucleophiles was always at least 10-fold excess. All reactions were finished within 10 seconds. Observed kinetics data confirmed the reversible reaction pathway of the substitution (Scheme 2), where *k*_1_ is the rate constant for direct, while *k*_−1_ is the rate constant for the reversible reaction:

The pseudo-first-order rate constant, *k*_obs_, was calculated as an average value of six or seven independent kinetic runs (all data are given in Table S1, ESI†). The linear dependence of the pseudo-first-order rate constants as a function of the nucleophile concentration enables the calculation of the corresponding rate constants *k*_1_ and *k*_−1_, by eqn (1):

1 *k*_obs_ = *k*_1_[Nu] + *k*_−1_

Activation parameters (activation entropy – Δ*S*^≠^ and activation enthalpy – Δ*H*^≠^) are obtained by Eyring eqn (2) and presented in Table 7:2
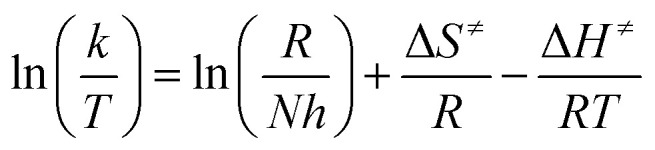


**Table 7:** Rate constants and activation parameters for the substitution reactions of complex F with the selected nucleophiles in Hepes buffer (pH = 7.2, 25 mM Hepes)

Nu	*λ* (nm)	*T* (K)	*k*_1_ × 10^4^ M^−1^ s^−1^	*k*_−1_ s^−1^	Δ*H*^≠^ kJ mol^−1^	Δ*S*^≠^ JK^−1^ mol^−1^
5′-GMP	253	288	1.19 ± 0.10	0.13 ± 0.07	19 ± 3	−116 ± 9
		298	1.68 ± 0.03	0.28 ± 0.10		
		308	2.17 ± 0.04	0.43 ± 0.05		
Guo	252	288	0.43 ± 0.08	0.41 ± 0.06	16 ± 2	−133 ± 7
		298	0.53 ± 0.05	0.53 ± 0.03		
		308	0.75 ± 0.02	0.75 ± 0.04		
Ino	248	288	1.09 ± 0.03	0.23 ± 0.02	9 ± 1	−151 ± 2
		298	1.26 ± 0.04	0.04 ± 0.03		
		308	1.52 ± 0.04	0.06 ± 0.01		

The lability of some copper(ii) complexes is already known,^42–44^ considering that the water exchange constant for [Cu(H_2_O)_6_]^2+^ has a value of ∼10^9^.^45^ This characteristic is connected with the octahedral geometry of copper(ii) complexes. The mechanism of this substitution process was confirmed as dissociative (*D*) or *I*_d_ mechanism.^45^

However, substitution reactions of copper(ii) complexes with square-pyramidal or trigonal-bipyramidal structures were also studied, and the obtained rate constants were in the range of 10^4^–10^6^.^45,46^

According to the values obtained within this investigation (∼10^4^), copper(ii) complex F is more reactive in comparison with square-planar [CuCl_2_(en)] or square-pyramidal [CuCl_2_(terpy)].^41^ The obtained order of reactivity of the nucleophiles is 5′-GMP > Ino > Guo. Namely, it is well known that the rate of nucleophilic substitution depends on the structure of the entering nucleophiles. Also, the coordination of these nucleophiles occurs *via* a nucleophilic attack on the N7 donor atom of the purine base. The higher reactivity of the nucleotide (5′-GMP) than nucleosides (Ino, Guo) can be explained by the presence of the electronic interactions confirming that the primary process involves partial pre-association of the metal complex with phosphate group in 5′-GMP.^47^

Therefore, substitution reactions were followed at three different temperatures in order to get insight into the mechanism of substitution. Since the values of the activation entropy for all reactions are negative (Fig. S1, ESI†), it can be concluded that the processes of substitution undergo *via* associative (*A*) or *I*_a_ mechanism, as published earlier.^45,46^

## Conclusion

A small series of newly synthesized and another small series of copper(ii) complexes that we synthesized earlier were used to examine the antitumor potential. The anticancer potential of copper complexes was tested *in vitro* against three tumor cell lines (HeLa, A549, and LS174T) and one normal cell line(MRC-5) using the MTT test. Importantly, after this test for the most active compounds, the mechanism of cytotoxic activity was examined in detail. In these tests, compounds B, D, and F showed the most potent cytotoxic activity against all tested malignant cell lines, while E and G showed significant reductive activities against A549 and MRC5 cell lines. Cell cycle analysis tests revealed that compounds D and F cause the increase in the percentage of HeLa cells in G2/M phases and the decrease in the G1 phase compared to untreated cells (control). The ROS level analysis revealed that compound D could achieve a potential anticancer effect by reducing ROS levels in the treated malignant cells. The DNA damage analysis showed that exposure to compound F caused DNA damage, which is significantly higher than the control group values (untreated cells). Analyzes of proteins related to hypoxia have shown that HeLa cells treated with compounds D and F decrease the expression of HIF-1α in a dose-dependent manner, suggesting their role as potential anticancer agents. Further investigations revealed that the obtained *K*_a_ values of BSA-D or F complexes are in line with the fact that our compounds have the appropriate capabilities for future use in clinical practice because they properly bind to BSA. Fluorescence quenching study of compound F with DNA indicated that the tested compound partially substitutes EB from the EB–DNA complex and interacts with DNA *via* intercalation, while obtained *K*_sv_ constant showed that the tested compound binds relatively strongly to DNA molecule. Kinetic investigations showed the best reactivity of 5′-GMP in comparison with Ino or Guo, which is connected with the phosphate group on that nucleophile, while the mechanism of substitution can be described as associative.

## Experimental section

### Materials

Inosine (Ino) and 2-[4-(2-hydroxyethyl)piperazin-1-yl]ethane-sulfonic acid (Hepes buffer) were ordered from Acros Organics. All the other used solvents and substrates were ordered from Sigma. The melting points (Mp) of the new compounds were calculated on a Mel-Temp apparatus and were not corrected. The IR spectra of novel compounds were recorded using PerkinElmer Spectrum One FT-IR spectrometer on a KBr pellet. Mass spectrometry is done using a Waters Micromass Quattro II triple quadrupole mass spectrometer and MassLynx software. Microanalyses of C, H, and N were performed on CarloErba EA1108. Fluorescence data were obtained using an RF-1501 PC spectrofluorometer (Shimadzu, Japan).

### Synthesis of copper(ii) complexes A, B and C

All novel copper(ii) complexes are synthesized in accordance with the previously published procedure.^24^

#### [Cu(2a)_2_H_2_O] (A)

Green powder; yield: 72%; mp = 147 °C; IR (KBr): *ν* 3294, 1741, 1666, 1552, 1544, 1392 cm^−1^; ESI-MS (*m*/*z*) = 686 [M + H]^+^. Calcd for C_32_H_34_N_2_O_11_Cu (%): C 56.01, H 4.99, N 4.08; found: C 56.12, H 4.97, N 4.05.

#### [Cu(2b)_2_H_2_O] (B)

Green powder; yield: 83%; mp = 183 °C; IR (KBr): *ν* 3310, 1724, 1644, 1508, 1255 cm^−1^. ESI-MS (*m*/*z*) = 818 [M + H]^+^. Calcd for C_40_H_38_N_2_O_13_Cu (%): C 58.71, H 4.68, N 3.42; found: C 58.66, H 4.70, N 3.41.

#### [Cu(2c)_2_H_2_O] (C)

Green powder; yield: 68%; mp = 237 °C; IR (KBr): *ν* 3506, 3446, 1726, 1637, 1566, 1506, 1250 cm^−1^. ESI-MS (*m*/*z*) = 784 [M + H]^+^. Calcd for C_42_H_40_O_11_Cu (%): C 64.32, H 5.14; found: C 64.43, H 5.11.

### Cell lines

To evaluate the cytotoxic potential of copper complexes we conducted an experiment on one normal, human embryonic lung fibroblast (MRC-5) cell line and three cancer cell lines: human cervical adenocarcinoma (HeLa), human lung adenocarcinoma (A549) and human colon adenocarcinoma (LS174T). Standard conditions RPMI-1640 medium supplemented with 3 mM l-glutamine, 100 μg per mL streptomycin, 100 IU per mL penicillin, 10% heat-inactivated (56 °C) fetal bovine serum, and 25 mM Hepes adjusted to pH 7.2 with a bicarbonate solution, the temperature of 37 °C, the atmosphere of 5% CO_2_ and humidified air were used for cell grow cells. Cells were purchased from the American Type Culture Collection (Manassas, VA, USA), while RPMI 1640, l-glutamine, and Hepes were purchased from PAA (Pasching, Austria).

### MTT assay for cell viability

To assess the effect of the tested copper complexes on cell viability, we conducted the MTT assay (3-(4,5-dimethylthiazol-2-yl)-2,5-diphenyltetrazolium bromide – MTT), previously described by Mosmann^48^ and modified by Mitsuharu and Abe.^49^ This reductive assay is based on the reduction of yellow tetrazole, MTT, to purple formazan crystals by cellular reducing agents, which corresponds to cell viability. The formazan concentration in the cells correlates to the degree of light absorption.

Cells were seeded in 96-well plates at: 3000 cells per well for HeLa, 5000 cells per well for MRC-5 and A549, and 7000 cells per well for LS174T. After 24 h, cells were treated with five different concentrations of the tested complexes. After 72 h of incubation, 20 μL of MTT solution (5 mg mL^−1^ of phosphate-buffered saline, PBS) was added, and samples were further incubated for an additional 4 h at 37 °C in a humidified atmosphere of 5% CO_2_ (v/v). Then, 100 mL of 100 g L^−1^ sodium dodecyl sulfate (SDS) was added. On the next day, 24 h later the absorbance (*A*) was measured at 570 nm, 24 h later.

The concentration of the complexes that inhibits cell survival by 50% compared to the control corresponds to the obtained IC_50_ value. All experiments were performed in triplicates.

### Cell cycle analysis by flow cytometry

For cell cycle analysis, HeLa cells were treated with copper complexes for 24 h and 48 h (in concentrations IC_50_ and 2IC_50_). After the incubation, the cells were collected, washed with PBS, and fixed in 70% ethanol, as described in the previously described protocol.^50^ Collected samples were stored at −20 °C for at least one week before staining. After one week, HeLa cells were defrosted, collected by centrifugation, washed and resuspended in PBS containing RNase A, and incubated for 30 min at 37 °C. Then, the staining of cells was performed with propidium iodide. The evaluation of the cell cycle of treated HeLa cells was done using a BD FACS Calibur flow cytometer and CELL Quest software.

### Evaluation of ROS production

The level of ROS in treated HeLa cells was observed after 24 h of incubation with subtoxic IC_20_ concentrations of tested copper complexes. According to the previously described method, upon the treatment, cells were collected, washed with PBS, and incubated with 2′,7′-dichlorodihydrofluorescein diacetate (30 μM in PBS) for 45 min at 37 °C. Afterward, cells were rewashed with PBS and analyzed.^51^ The fluorescence intensity emitted by the dichlorofluorescein was determined on a BD FACS Calibur flow cytometer and analyzed using CELLQuest software.

### Evaluation of DNA damage by Comet assay

The cells were treated with IC_20_ concentrations of selected copper complexes for 24 h. Then, the cells were collected, washed with PBS, suspended in a freezing medium (RPMI with 10% DMSO and 20% FCS), and frozen at −80 °C. We have evaluated DNA damage by the previously described version of the single-cell gel electrophoresis assay.^52^ Frozen cells were thawed by the addition of 1 mL of PBS to an aliquot of 0.5 mL and centrifuged for 10 min at 2000 rpm at 4 °C. The washing procedure was repeated with a pellet. The concentration of cells was adjusted to 2.5 × 10^5^ per mL with PBS and 30 μL of cell suspension was mixed with 140 μL of 1% LMP agarose at 37 °C. Then, 10 μL of agarose-cell suspension was placed on an NMP agarose-coated slide. Then, cells were lysed for 1 h at 4 °C with a buffer containing: 2.5 M NaCl, 0.1 M Na_2_EDTA, and 10 mM Tris with 1% Triton X-100 pH 10. Subsequently, horizontal gel electrophoresis at a voltage gradient of 1 V cm^−1^ across the platform at 4 °C was performed in a proper solution (0.3 M NaOH, 0.001 M Na_2_EDTA) (20 min incubation and 30 min of electrophoresis). Neutralization of the slides was done by washing them for 10 min in PBS at 4 °C. Then, they were fixed with 70% ethanol for 10 minutes, incubate for 10 min in absolute ethanol, and stained with SYBR Gold (Invitrogen) for 30 min in the dark. Stained slides were washed twice with water and dried in the dark. Scoring of the comets was carried out using a semi-automated image analysis system (Comet Assay IV; Perceptive Instruments). Fifty nucleoids per gel were analyzed, and the results were expressed as a percentage of tail intensity (% of DNA in the tail).

### Evaluation of HIF-1α and PDK-3 expression by western blot

Human cervical carcinoma cells were seeded at a density of 0.5 × 10^6^ cells per well onto six-well plates and incubated at 37 °C for 24 h and treated with copper complexes (concentrations of IC_50_ and IC_20_). After 24 h treatment, cells were washed three times with ice-cold PBS, collected, and centrifuged at 3000 rpm for 5 min. Then cells were resuspended in RIPA buffer supplemented with cOmplete™, EDTA-free Protease Inhibitor Cocktails, and incubated on ice for one hour. Afterward, cells were sonicated (three cycles: 10 s of sonication followed by cooling for 30 s), and whole-cell lysates were put on ice for one hour and centrifuged at 11 000 rpm for 20 min, at 4 °C. Then, supernatants were collected and protein concentrations were determined with the BCA protein assay kit (Thermo Fisher Scientific, Waltham, Massachusetts, USA). Extracted proteins were mixed with loading buffer, denatured for 10 min at 90 °C, loaded into 10% Tris-Glycine gels, and separated by the standard SDS-PAGE. After separation, proteins were transferred to nitrocellulose membranes, and membranes were blocked with 5% nonfat milk in TBST buffer containing 0, 1% Tween 20 for one hour at room temperature. An overnight incubation followed, at 4 °C in primary antibody [anti – PDK3 (1 : 1000, ab 154549, Abcam, Cambridge, UK); anti – HIF-1α (1 : 300, sc – Santa Cruze Biotechnology, Heidelberg, Germany); anti – β-actin (1 : 1000, ab 3280, Abcam, Cambridge, UK)]. Afterward, membranes were rinsed three times for 10 with PBST buffer, and incubated with appropriate HRP-conjugated secondary anti-rabbit or anti-mouse antibody (Lumi – lightPLUS western blotting kit, Roche Applied Science, Penzberg, Germany) for 1 hour at room temperature and washed again in TBST buffer. Visualization was performed by chemiluminescence (Lumi – lightPLUS western blotting kit, Roche Applied Science, Penzberg, Germany) with ChemiDoc Imaging System (BioRad, Hercules, CA, USA). The intensities of immunoreactive bands were determined using Image Lab 6.0.1. software (BioRad, Hercules, CA, USA). Protein loading was normalized to β-actin.

### Kinetic measurements

In order to find the appropriate wavelength at which kinetic experiments can be performed, UV-vis spectra of the mixture of complex and nucleophile solutions were recorded in the range between 200–600 nm. Working wavelength is described as a wavelength that displays the highest change in the absorbance during time. The values of the working wavelengths are given in Table 7. The same volumes of the solutions of copper(ii) complex (concentration 0.01 mM in 25 mM Hepes buffer) and the nucleophile (Guo, Ino or 5′-GMP, concentration in the range 0.05–0.2 mM in 25 mM Hepes buffer) were mixed directly in “stopped-flow” instrument and the interactions were followed within 10 seconds. The pseudo-first-order rate constants, *k*_obs_, were obtained by monitoring the absorbance changes with time at working wavelength, like the average value of six or seven independent kinetic runs. Kinetic traces are given in Fig. S2, ESI.† The substitution reactions were followed at three different temperatures (288, 298, or 308 K) to determine the substitution mechanism. The computational programs Pro Data SX, OriginPro 2016, and Microsoft Excel 2007 were used to get calculations.

## Conflicts of interest

There are no conflicts to declare.

## Supplementary Material

RA-012-D2RA05797B-s001
