# Supplementary material for: Synthesis, characterization, antitumor potential, and investigation of mechanism of action of copper(ii) complexes with acylpyruvates as ligands: interactions with biomolecules and kinetic study
Source: RSC Adv. 2022 Oct 26;12(47):30501–13. doi: 10.1039/d2ra05797b (PMC9597287; doi:10.1039/d2ra05797b)
Supplement: RA-012-D2RA05797B-s001 [file RA-012-D2RA05797B-s001.pdf]

Electronic Supplementary Material (ESI) for RSC Advances.
This journal is © The Royal Society of Chemistry 2022

Synthesis, characterization, antitumor potential, and investigation of mechanism of action of copper(II) complexes with acylpyruvates as ligands: Interactions with biomolecules and kinetic study

Nenad Joksimović,[a*] Jelena Petronijević,[a] Snežana Radisavljević,[a] Biljana Petrović,[a] Kristina Mihajlović,[a] Nenad Janković,[b] Emilija Milović,[b] Dušan Milivojević,[c] Bojana Ilić,[d] Ana Djurić[e*]

[a]University of Kragujevac, Faculty of Science, Department of Chemistry, Radoja Domanovića 12, 34000 Kragujevac, Serbia. E-mail: nenad.joksimovic@pmf.kg.ac.rs

[b]University of Kragujevac, Institute for Information Technologies Kragujevac, Department of Sciences, Jovana Cvijića bb, 34000 Kragujevac, Serbia

[c]Vinča Institute of Nuclear Science University of Belgrade, P.O. Box 522, 11001, Belgrade, Serbia

[d]Clinic for Endocrinology, Diabetes and Metabolic Diseases, University Clinical Centre of Serbia, Belgrade, Serbia

[e]Institute of Oncology and Radiology of Serbia, Pasterova 14, 11000 Belgrade, Serbia

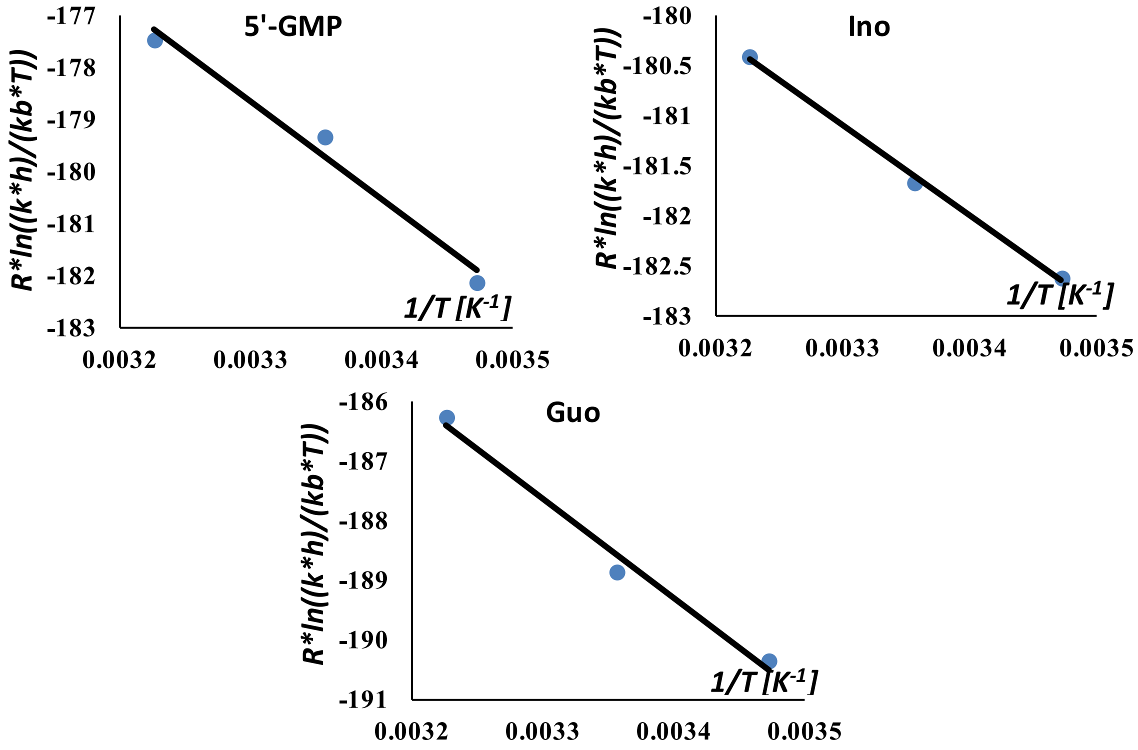

Fig. S1. Eyring plots for the reactions of copper(II) complex **F** with different nucleopohiles in Hepes buffer (pH=7.2, 25mM Hepes).

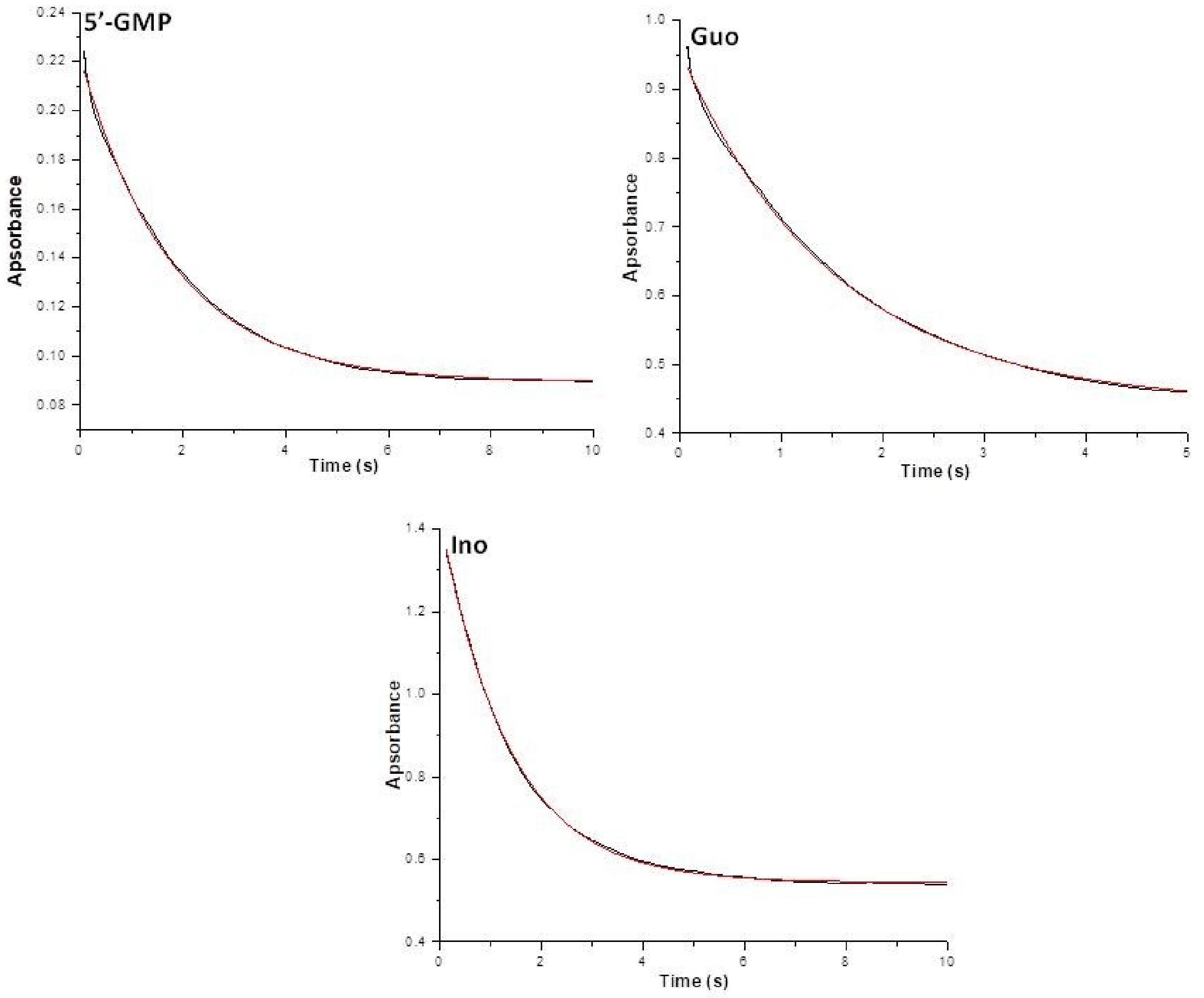

Fig. S2. Kinetic traces for the substitution reactions of copper(II) complexes and the nucleophiles.

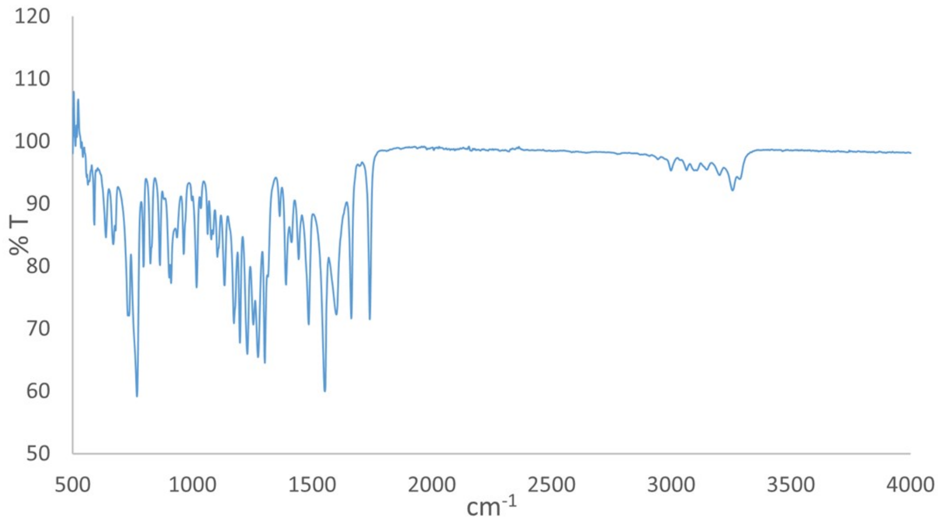

Fig S3.IR spectra of compound **A**

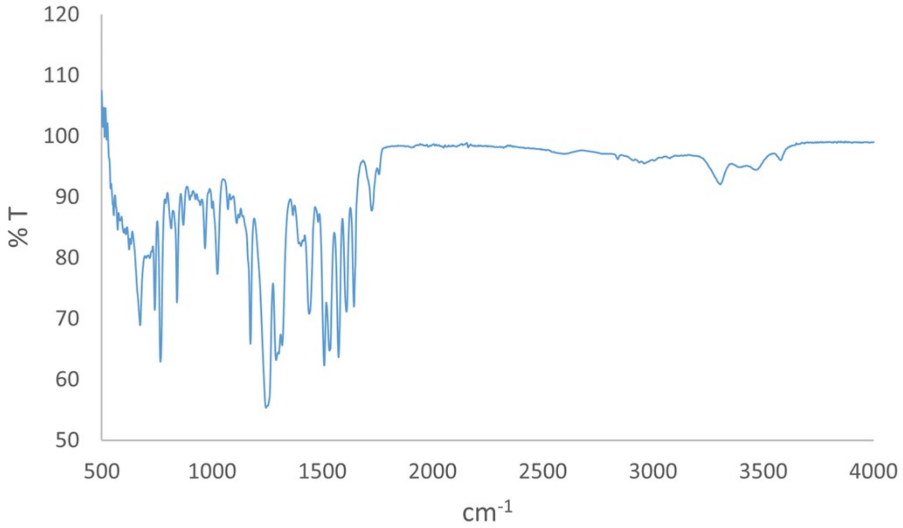

Fig S4.IR spectra of compound **B**

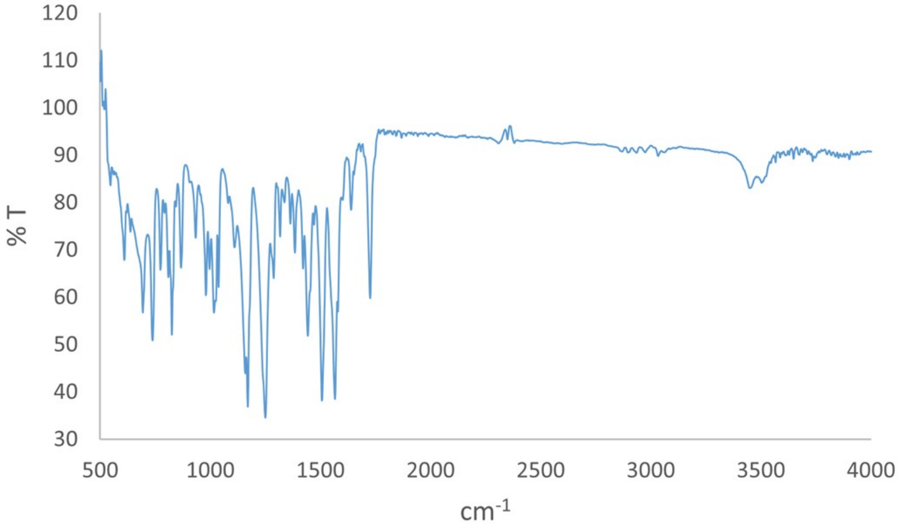

Fig S5.IR spectra of compound **C**

Table S1. Observed *pseudo*-first order rate constant as a function of the nucleophile concentration and temperature for the substitution reactions in 25mM Hepes buffer (pH = 7,2).

T/K	10^{-5} C_L/M	k_{obs}/s^{-1}		
		**5'-GMP**	**Guo**	**Ino**
288	5	0.680(7)	0.512(6)	0.553(7)
	7.5	1.080(6)	0.633(7)	0.810(7)
	10	1.300(6)	0.840(6)	1.093(6)
	12.5	1.698(7)	1.071(7)	1.398(6)
	17.5	1.945(6)	1.261(7)	1.618(6)
	20	2.168(6)		
298	5	1.040(7)	0.681(6)	0.600(6)
	7.5	1.546(7)	0.793(6)	0.984(6)
	10	1.963(6)	1.050(7)	1.322(6)
	12.5	2.486(7)	1.285(7)	1.564(7)
	17.5	2.860(6)	1.630(6)	1.896(7)
	20	3.080(6)		
308	5	1.480(6)	0.944(7)	0.984(6)
	7.5	2.060(6)	1.171(7)	1.368(7)
	10	2.598(6)	1.490(6)	1.794(6)
	12.5	3.197(6)	1.873(7)	2.136(6)
	17.5	3.689(7)	2.285(6)	2.496(6)
	20	4.176(7)		
